# SynBiopython: an open-source software library for *Synthetic Biology*

**DOI:** 10.1093/synbio/ysab001

**Published:** 2021-02-22

**Authors:** Jing Wui Yeoh, Neil Swainston, Peter Vegh, Valentin Zulkower, Pablo Carbonell, Maciej B Holowko, Gopal Peddinti, Chueh Loo Poh

**Affiliations:** 1 NUS Synthetic Biology for Clinical and Technological Innovation (SynCTI), Life Sciences Institute, National University of Singapore, Singapore, Singapore; 2 Institute of Systems, Molecular and Integrative Biology, University of Liverpool, Liverpool, UK; 3 Edinburgh Genome Foundry, University of Edinburgh, Edinburgh, UK; 4 Instituto Universitario de Automática e Informática Industrial, Universitat Politècnica de València, Valencia, Spain; 5 Manchester Synthetic Biology Research Centre for Fine and Speciality Chemicals (SYNBIOCHEM), Manchester Institute of Biotechnology, The University of Manchester, Manchester, UK; 6 CSIRO Synthetic Biology Future Science Platform, Canberra, ACT, Australia; 7 VTT Technical Research Center of Finland, Espoo, Finland

**Keywords:** Software, Synthetic Biology, Biofoundries, Open-source, Automation

## Abstract

Advances in hardware automation in synthetic biology laboratories are not yet fully matched by those of their software counterparts. Such automated laboratories, now commonly called biofoundries, require software solutions that would help with many specialized tasks such as batch DNA design, sample and data tracking, and data analysis, among others. Typically, many of the challenges facing biofoundries are shared, yet there is frequent wheel-reinvention where many labs develop similar software solutions in parallel. In this article, we present the first attempt at creating a standardized, open-source Python package. A number of tools will be integrated and developed that we envisage will become the obvious starting point for software development projects within biofoundries globally. Specifically, we describe the current state of available software, present usage scenarios and case studies for common problems, and finally describe plans for future development. SynBiopython is publicly available at the following address: http://synbiopython.org.

## Introduction

With synthetic biology developing at an increasing pace, there are now a large number of tools covering the Design-Build-Test-Learn (DBTL) cycle available to researchers, originating from both academic and commercial sources. For instance at the Design stage, computer-aided metabolic engineering tools such as Cameo (1) and RetroPath2.0 (2); or tools in the transition from Design to Build for sequence optimization such as DnaChisel (3) and PartsGenie (4); tools at the Build stage such as CloneFlow for planning ligase cycling reaction DNA assemblies (5); tools at the Test stage, such as mzmine for mass spectrometry data processing (6), tools from Test to Learn and Design such as BioModel Selection System (BMSS) that performs automated BioModel selection (7), or tools facilitating the transition from Learn to Design such as the cobrapy library for genome-scale metabolic modeling (8). Increasing automation in synthetic biology laboratories [the consensus term for such an automated lab used for synthetic biology research and development is ‘biofoundry’ (see also ref. to GBA article, https://biofoundries.org/)] is posing another set of problems. While many laboratories may not require sophisticated software for data collection, sample tracking or batch genetic construct design, such software is essential for heavily automated labs. The main reason for this is the significant number of samples being processed daily (in the order of 10^2^ to 10^4^). Without appropriate software, manually generated mistakes can become increasingly prevalent and, given the volume of samples processed, such mistakes can become very costly in terms of both time and money.

Selecting appropriate software solutions for an automated laboratory can be difficult. The solutions are scattered and there are no definite guidelines or universally agreed state-of-the-art. As a result, many groups have created software solutions in-house, which typically involves directly hiring developers. This approach, however, leads to the multiplication of efforts, and since these solutions are usually developed with that specific lab in mind, it is often difficult to reuse these solutions in a different lab, even if the code is open-sourced. Commercial solutions, on the other hand, are usually developed with big operations in mind and do not scale well to smaller operations. Additionally, such solutions are often expensive and such costs are hard to justify for a relatively small, albeit automated, lab. Furthermore, many tools, whether academically or commercially developed, are typically end-to-end applications. Such solutions provide a predefined set of functionalities, which are difficult for other developers to unpick in order to reuse individual components in their own software.

In the 2000s, the bioinformatics community was in a similar situation and created Biopython (9) as a library of primitives. The advantages of such libraries include, (i) increased reliability, due to community testing; (ii) increased reusability and interoperability between the different modules of the project; and (iii) increased community uptake, due to easier discovery of features that are organized under a single umbrella project. The approach has been very successful, with over 2000 manuscript citations and 3500 Github projects using Biopython. However, since Biopython is primarily focused toward classical bioinformatics, with an emphasis on sequence analysis, the Global Biofoundries Alliance (GBA) (10) software group identified a need for a library specific to the requirements of the synthetic biology community. These requirements include tools assisting in DNA design and assembly, software for automation and robotic equipment. A project specific to synthetic biology provides better visibility and also encourages contributions from developers in this field.

The Software Working Group of Global Biofoundries Alliance, therefore, introduces a new package, named *SynBiopython*, to support aspects of development efforts that are common to many DNA design and assembly projects. Python is recognized as being ubiquitous in biofoundry software development efforts and is, therefore, a natural choice for such a consolidated, collaborative effort.

In introducing this work, it is recognized that there remains a large amount of development work to be performed, requiring the introduction of a multitude of new modules, for the package to be considered ‘full-suite’. This article, therefore, acts as a ‘call to arms’ on the synthetic biology community, introducing the concept of reusable libraries, exemplifying its use through the development of specific, community-developed modules and specifying the governance requirements to manage the growth of the resource over time.

The initial modules demonstrated here include standard file parsers and tool interoperability, an automation library and support for codon usage tables. The presented tools were chosen from a number of tools that were originally written in different biofoundries that are part of the GBA. The decision was made to work on these modules first to meet the following general objectives of SynBiopython: (i) collation and development of synthetic biology-oriented code and tools in Python; (ii) support for both novice and advanced developers of synthetic biology software; and (iii) prevention of duplicated efforts. There are also a number of specific aims that the Authors would like SynBiopython to meet: (i) standardization of read/write operations and other procedures and automation related tools to allow ease of access and interaction; (ii) simplification of parsing of different synthetic biology-related file formats; and (iii) development of more intuitive APIs and wrapper functions on top of more complex code, hiding underlying details.

## Results

Here, we describe the three modules that show how future modules in SynBiopython should be written and used. Each module is provided together with a case study and some example code for easier understanding. First, we describe Genbabel, a tool that enables translation between file formats relevant to synthetic biology. Next, we discuss the Automation Library, a module that can be used to create instruction files for automated equipment. Finally, we show how the Codon Usage Tables tool can be used to optimize DNA sequences.

### Standard file parsers and tool interoperability: Genbabel

Driving interoperability between tools via a common standard is a deep-rooted effort in synthetic biology. Several standard file formats such as GenBank (11), FASTA, Synthetic Biology Open Language (SBOL) (12), Systems Biology Markup Language (SBML) (13), Simulation Experiment Description Markup Language (SED-ML) (14) and Computational Modeling in Biology Network (COMBINE) archives (15) have been proposed at different information levels to overcome the reproducibility challenge and to serve as a common integrated knowledge base for data sharing. Despite these standards having been adopted in many of the developed tools, there is no one-size-fits-all tool that supports the parsing of these common standard files in the field of synthetic biology. To mitigate these issues, SynBiopython introduces a universal environment, named Genbabel, which serves as a repository of standard file parsers built upon existing libraries and applications to enable easy generation and conversion of different standard files as mentioned above, including formats for DNA/protein sequences, genetic circuits, and model simulations. This aims to reduce redundant or overlapping efforts and to improve reusability which are essential to accelerate the progress of the field.

At the lower level, GenBank and FASTA files are the most ubiquitous standard formats used to encode DNA and protein sequence data (11). To capture the structural information at a higher level, the SBOL and SBOL Visual compliant diagrams have been introduced and applied in many software platforms (12). To enable the transferability of different data standards, built upon existing online platform and packages (16–18), a standard file parser has been developed in Genbabel to support the conversion between SBOL files and the aforementioned sequence data formats including General Feature Format (GFF3), and the rendering of highly customizable genetic circuits and their associated regulations.

Aligning with the use of the model-driven approach in forward and reverse cell engineering, the advent of SBML enables the representation of computational models in a declarative form to ease the exchange of quantitative descriptions (13). SBML is widely used for modeling and simulation for chassis optimization through Python-based tools for flux analysis and knock-out/knock-in optimization such as COBRApy (8) or cameo (1). Genome-scale metabolic models for the most common industrial hosts are available at public databases, such as BioModels (19) and BiGG (20) and can be downloaded in SBML. The support of modeling in general and SBML in particular is therefore of increasing interest to the synthetic biology community. The Genbabel module was thus extended to provide the capability of generating SBML files and other formats related to modeling, such as SED-ML (14) and COMBINE archives in Open Modeling EXchange (OMEX) format (15), which are hinged on several developed packages (21–23).

The incorporation of Genbabel module in the SynBiopython package seeks to provide a universal parser environment which supports the gathering of and interfacing with parsers spanning across gene sequence, circuit, and systems levels. Longer-term goals include the development of an improved interface, linking file parsing encoded in different formats from sequence, structure, model, simulation and analysis.

### Case study: standard file generation and model generation

**Figure 1.**
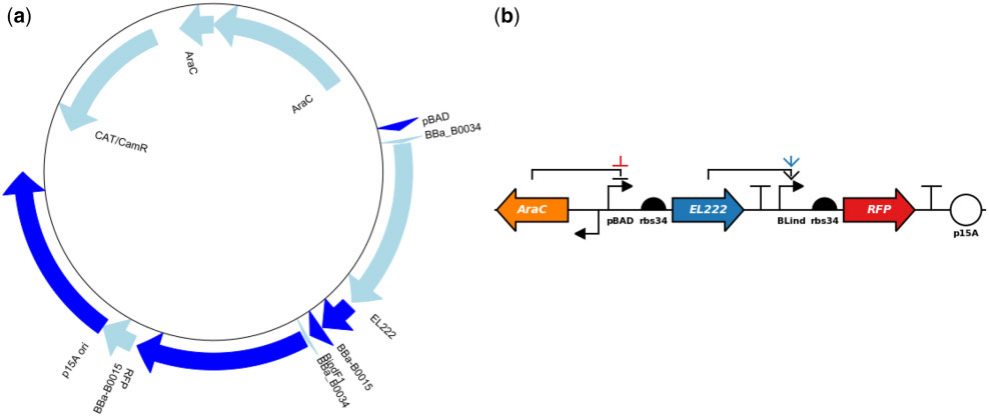
A case study of an AND logic gate genetic circuit generated using the Genbabel module. The AND gate consists of a blue-light inducible system using a photosensitive DNA-binding protein EL222 (24, 25). The system is turned on in the presence of both blue light and the arabinose inducer, to drive the expression of red fluorescent proteins (RFPs). (**a**) Circuit plasmid map generated using the GenSBOLconv submodule; and (**b**) SBOL Visual compliant gene circuit diagram generated using the SimpleDNAplot submodule of Genbabel module.

To demonstrate the capability of Genbabel, we present an example (Code Block 1) to demonstrate the conversion of a GenBank file, which encodes an AND logic gate genetic circuit generated from Benchling (Benchling Inc., San Francisco, USA) during the Design phase, to SBOL file using the GenSBOLconv submodule. The circular plasmid map can also be constructed based on the given GenBank file (Figure 1a). Meanwhile, the corresponding SBOL-compliant genetic circuit diagram can be generated using the SimpleDNAplot submodule (Figure 1b).

**Figure 2.**
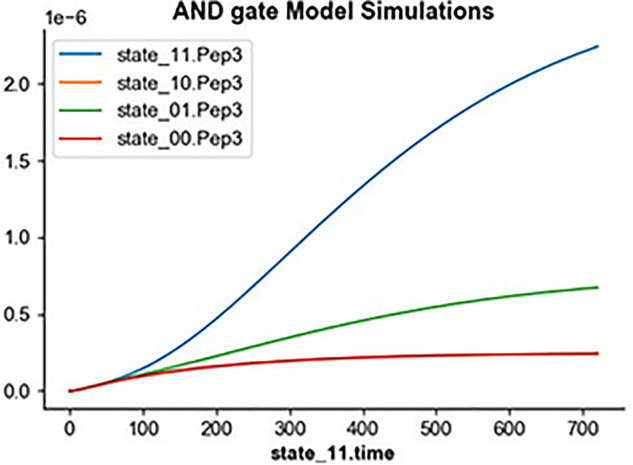
Model simulation results for the AND logic gate with four state inputs (00, 01, 10, 11) generated from the SBML and SED-ML files contained in the COMBINE archive OMEX format. The simulation is performed using the execute_inlineomex function from the SEDMLOMEXgen submodule.

During the Design phase before the actual circuit construction, one can also utilize the SBMLgen submodule to generate the SBML file which encodes the kinetic model of the AND gate for simulation as demonstrated in Code Block 2. All the different elements such as the ODEs, variables, initial conditions, parameters names, values and units are to be provided in lists of strings as input arguments to the export_sbml function. Otherwise, in the Learn phase, using characterization data of the AND gate, an SBML file can also be generated via running the BMSS tool (7). With the available SBML file, the corresponding SED-ML file and COMBINE archive in OMEX format can subsequently be generated and executed using the SEDMLOMEXgen submodule with the AND gate simulation results shown in Figure 2. These formats ensure the reproducibility of the model implementation and simulation. A detailed example code implementation is provided in the SynBiopython GitHub repository.

### Automation library

In the spirit of developing computational infrastructure across the DBTL cycle, the Build phase is supported through the introduction of the SynBiopython automation library. The goal of the library is to provide an easy-to-use, standardized solution for the creation of automated workflows for biofoundries. It is envisaged that the library will act as the first software suite that a user of a biofoundry will have contact with and by setting good practices it will reinforce them in the users.

There are currently a number of solutions that allow lab workflow automation, including Aquarium (26), Antha (Synthace Ltd., London, UK), TeselaGen BUILD (TeselaGen Biotechnology Inc., San Francisco, USA) and the Autoprotocol [Strateos Inc. (formerly Transcriptic), Menlo Park, USA]. However, these may not be suitable for a biofoundry operator. Some of these are still under development, or do not allow the development of protocols via scripts. Proprietary software can be costly and inhibits collaborative development and adapting the software to custom needs.

The GBA recognizes automation to be a major bottleneck in the development of the biofoundry technology. For the most part, each lab uses their own collection of open-source, in-house and commercial automation software which makes collaborations and comparative studies very difficult. Many of the routine tasks that are performed in biofoundries involve liquid manipulation, including dilutions, normalizations, transfers between plates and rearraying. Creating reliable protocols and picklists for such operations is a time-consuming effort and, without proper software support, very error prone.

The introduction of the lab automation module within the SynBiopython package aims to address these issues. This module, adapted from Plateo by the Edinburgh Genome Foundry, enables generation of picklists and protocols for commonly used machines, focusing on liquid handlers (e.g. Labcyte Echo). A long-term goal is the integration of this module with a number of open-source libraries and APIs (e.g. Biopython, Benchling, Teselagen, other common LIMS or DNA synthesis providers) to enhance its data-tracking capability.

### Case study: generating a picklist

The lab automation module includes a number of classes: the plate class (e.g. a microplate) which contains objects of the well class (which stores information about the contents of a given well in the plate), the transfer class that stores information about transfers to be performed between wells and finally the picklist class, which contains a list of transfers to be made within a single plate or between different plates.

The example in Code Block 3 shows how a picklist can be generated. The picklist object can be initiated with a predetermined list of transfers to be performed or the transfers can be directly defined using the add_transfer method. After defining all the transfers, the picklist can be then translated to a form accepted by a relevant liquid handler (which will be a future feature) and finally executed. More detailed code demonstrating these features is available in the examples directory of the code repository.

### Codon usage tables

A typical task in the Design step of a biofoundry workflow is the optimization of the coding sequence of a given amino acid sequence for recombinant expression in a host of interest. As codon usage differs across organisms, such codon optimization is reliant on codon usage tables, which specify a given organism’s frequency of use of each degenerate codon. While codon usage tables are publicly available (27), there remains as yet no standardized means for the programmatic access and manipulation.

SynBiopython consequently includes a module for support of codon usage tables and codon optimization. This module is based on previous work from the Manchester Centre for Synthetic Biology (SYNBIOCHEM) and the Edinburgh Genome Foundry. Following a simple interface, codon usage tables may be automatically accessed from the Kazusa Codon Usage Database and used in a number of codon optimization methods. The library complements the existing Biopython CodonTable module but includes codon frequency in addition to translation tables. Example code for the codon module is provided in Code Block 4.

Future work may include support for custom codon usage tables of novel or rare organisms, more sophisticated codon optimization algorithms, and support for Biopython sequences.

### Future directions

It is hoped that future directions of development for the SynBiopython library will be driven by the needs of the community, and by interested volunteers who would happily provide useful modules that would be of general utility. Synthetic biology is an umbrella term, encompassing a number of sub-disciplines, and the SynBiopython project aspires to support a range of tools and applications across these numerous sub-communities.

One such application of interest for the metabolic engineering community is to provide a straightforward scripting way for *in silico* prototyping of genetic constructs once inserted into the optimized chassis organisms. Such approach should be addressed effortlessly, as examples and tutorials exist in both cameo and COBRApy about adding biochemical species and reactions to genome-scale models in order to represent the genetic circuit of metabolic circuits and pathways.

To connect the dots, combinatorial genetic circuits represented in SBOL and designed through tools such as Cello (28) should generate annotated SBML models that can be seamlessly added into the genome-scale model of the chassis. Standard interconversion procedures exist between SBML and SBOL and have been implemented in the Java-based iBioSim tool (29). In general, the generation of an annotated SBML model from SBOL can be accomplished by using terms from ontologies (30). Ontologies are controlled vocabularies that can be associated with different elements in the SBOL. The Sequence Ontology (31) allows defining roles to the components such as promoters, coding sequences or terminators, while the Systems Biology Ontology (SBO) (32) allows the definition of biochemical species and reactions. As a first approach, SBOL should provide the minimal annotations required in order to be able to integrate the engineered circuit of the pathway into the SBML model and perform steady-state flux analysis simulations.

**Figure 3.**
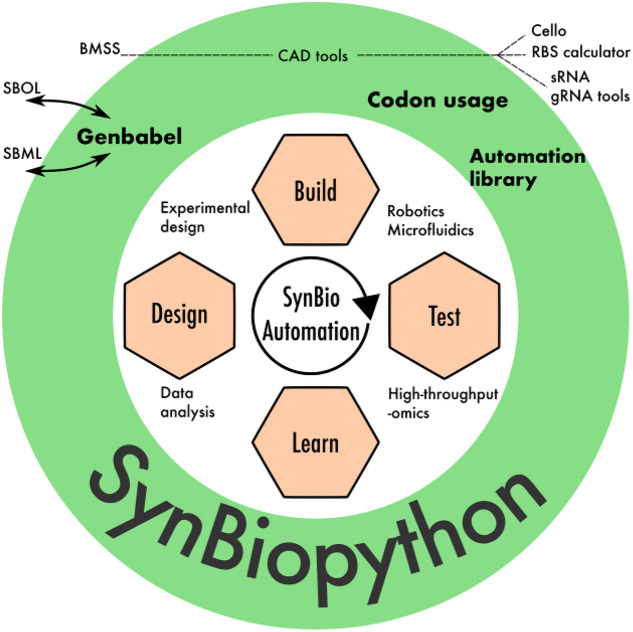
Current SynBiopython modules within the Design-Build-Test-Learn (DBTL) cycle of synthetic biology (33). Genbabel module provides the link between Design and Build by allowing the interconversion of sequence-based files into gene circuit representation format in SBOL, and the generation of SBML models and other modeling-related formats, which could then be interfaced through the CAD tools with external tools such as BMSS, Cello, RBS Calculator or sRNA, gRNA tools. The link between Build and Test is implemented through the Codon usage and the Automation library.

Furthermore, an extensive number of freely available online (web-based) and offline tools are available to expedite the different phases of the DBTL cycle of synthetic biology. Supporting interoperability between these tools will allow for more efficient development of computational pipelines and the reduction of redundant efforts (Figure 3). Such interoperability will pave the way toward a long-standing goal of synthetic biology: full lab automation assisted by streamlined computer-aided tools. Several useful tools that serve to automate design, modeling and optimization phases are compiled below, and depending upon the priorities of the community, these will be incrementally supported by future developments of SynBiopython. Such a platform for the support of third-party applications will be made highly extensible to allow more tools to be interfaced over time.

**Code Block 1.**
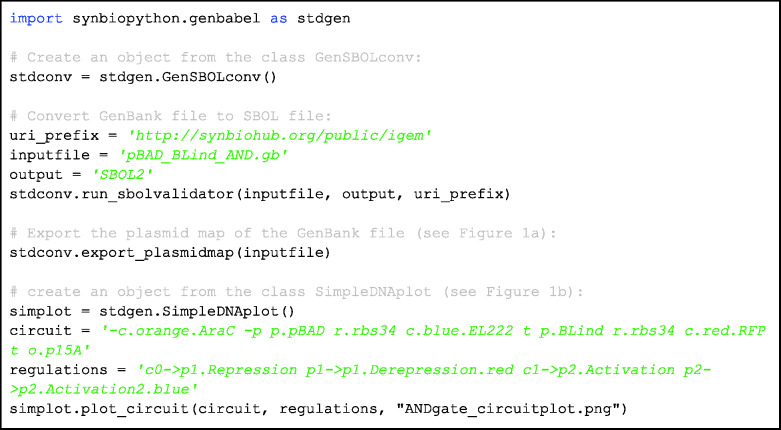
Demonstration of the features of GenSBOLconv and SimpleDNAplot submodules. Here, we exemplified the conversion of a GenBank file to SBOL file, which encodes an AND logic gate for a blue-light inducible system, using the run_sbolvalidator function from GenSBOLconv class. This function enables the interconversion of GenBank, Fasta, GFF3 and SBOL files. With the provided GenBank file, the linear and circular plasmid maps can be exported. Users can also employ the plot_circuit function from SimpleDNAplot class to generate the SBOL-compliant gene circuit diagram. The circuit configuration and the corresponding regulations were to be defined in the form of string following proper sequences separated by spaces. The alphabets p, r, c, t, o represent the promoter, ribosome binding site, coding sequence, terminator and origin with the negative sign denoting the reverse direction. Each of the parts consists of the part type followed by the color (optional) and part name (optional). The regulations were defined in the form of ‘from part->to part’ followed by the type of regulation and color (optional). The parts were numbered starting from 0 following the sequences defined in circuit from left to right.

**Code Block 2.**
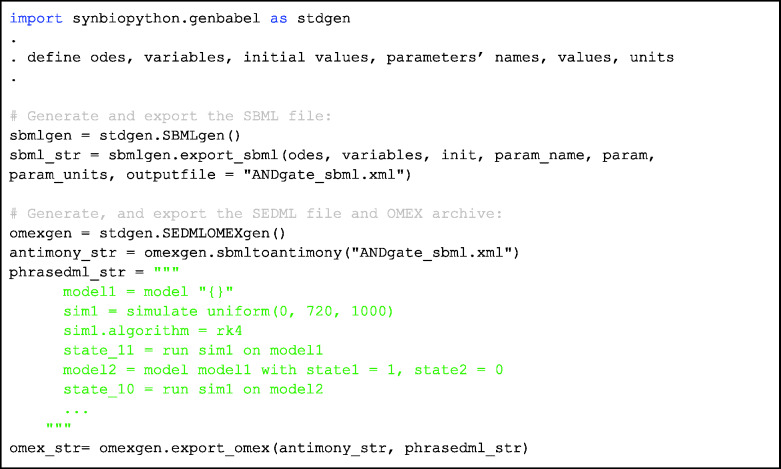
Demonstration of the features of SBMLgen and SEDMLOMEXgen submodules. To generate a SBML file, the function export_sbml from the SBMLgen class is used to generate the ANDgate_sbml.xml file. Input arguments such as ODEs, variables, initial conditions, parameters’ names, values and units have to be defined and provided into the function. To generate the COMBINE omex file, the previously generated SBML file is read and converted into an antimony string representation using function from SEDMLOMEXgen submodule. Users can then define the phrasedml string which encodes the descriptions for the simulation experiment. The antimony and the phrasedml strings are then supplied as input arguments to the export_omex function to generate the corresponding omex file.

**Code Block 3.**
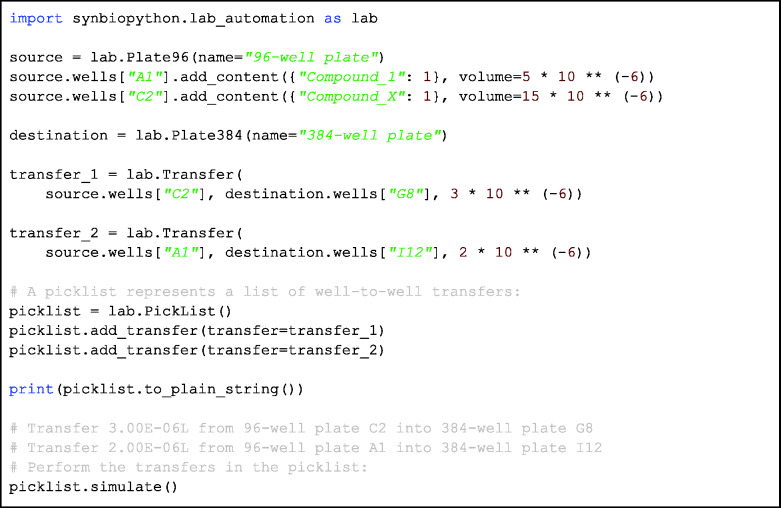
Demonstration of the lab automation module. First, a 96 well source plate object is created, followed by two lines which fill the wells of that plate with content of given volume (plate is created empty). Next, a 384 well destination plate is created, then two transfers from source wells to destination wells are defined. Finally, a picklist is created, and the transfers are added to it. The user can then choose to simulate the picklist to see if the transfers are resolved correctly.

Potential tools for future incorporation are as follows, and readers are encouraged to contact the Authors with comments regarding their prioritization and to make further suggestions.

- ‘Cello’ allows for the automatic design of genetic logic gates using a high-level language known as Verilog. Circuit performance can be predicted, factoring in growth and load (28).
- The ‘RBS Calculator’ predicts translation initiation rates, based on the start codon of mRNA transcripts, and designs and optimizes synthetic ribosome binding site (RBS) sequences to achieve a desired translation rate (34).
- The ‘Biomodel Selection System (BMSS)’ automatically derives or selects the best mathematical model based upon part/circuit characterization data (7).
- sRNA design tools include ‘IntaRNA’ which is used to predict the mRNA target sites for a given sRNA or to predict the interactions between two RNA molecules. ‘CopraRNA’ is built upon IntaRNA and computes whole-genome sRNA target predictions for a set of given organisms (35).
- gRNA design tools. ‘Cas-OFFinder’, ‘CHOPCHOP’ and ‘CRISPOR’ are free web-based tools which allow off-target site analysis, with some providing specificity scores and cleavage likelihood of a gene sequence (36).

The overarching goal is to support the interoperability of file formats and software tools, from sequence design, through automation, data analysis and representation, and machine learning, allowing for the development of computational pipelines across the DBTL cycle, complementing the work conducted on the bench.

## Conclusion

This work introduces SynBiopython, in which initial efforts in creating a standardized, open-source, Python library to be used in biofoundry-type facilities around the world are demonstrated. The library is modeled on the existing Biopython library, being divided into modules of different functionalities. To our knowledge, this is the first synthetic biology specific software package for standardizing development efforts across automated synbio facilities.

It is strongly envisaged that SynBiopython will be a community effort. As the global biofoundry community grows and more labs join the automation effort, the hope is to attract more developers and other stakeholders. A key goal is for members of the community to offer additional modules, used locally in their own labs but with perhaps wider utility, and thereby to help with the development and curation of the package. Such an approach has many mutual benefits, reducing duplication of efforts and thereby freeing up resources to focus on the development of more novel and innovative methods. It is clear that there are developers and users in the general synbio community with skills and interests that would benefit the development efforts of the SynBiopython package, and interested members are encouraged to mail info@synbiopython.org to discuss their potential involvement.

With an increasing number of contributors, a governance model will also be developed to help steer the future development of the package. Such governance matters include deciding on the scope of the package and which new modules to prioritize, and more technical matters including code standardization, automated testing and documentation requirements. All such decisions will be made with the consultation of the SynBiopython development community and more details can be found in the relevant file in the Github repository.

With the introduction of the SynBiopython package, a clear mechanism for the sharing and reusability of code being developed in individual biofoundries is proposed. Promoting such standardization and interoperability is not intended to stifle innovation, but rather to support the development of novel approaches through reducing effort spent on finding solutions to universal problems that are shared across many labs. Such developments are of benefit to all stakeholders in synthetic biology, from lab-based researchers, informaticians, research leaders and funders.

**Code Block 4:**
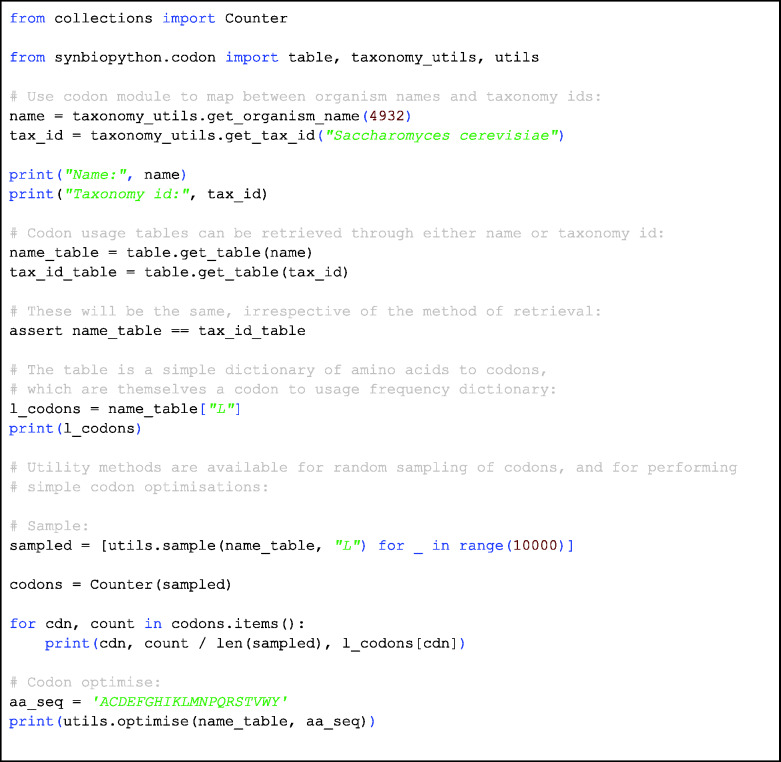
Demonstration of the features of the Codon Usage module, codon. The taxonomy_utils module supports mapping between organism names and taxonomy ids. The names and the taxonomy ids can be used to retrieve the codon usage table which is a simple dictionary of amino acids to codons, and the codons are themselves a dictionary of a codon to usage frequency.

## Code availability

The code repository for SynBioPython is located on Github: https://github.com/Global-Biofoundries-Alliance/SynBioPython
